# A Chromosomal-level genome assembly and annotation of fat greenling (*Hexagrammos otakii*)

**DOI:** 10.1038/s41597-025-04368-3

**Published:** 2025-01-15

**Authors:** Li Li, Fawen Hu, Dong Liu, Xiaolong Wang, Jing Diao, Yijing Zhu, Fengxiang Gao, Ying Fan, Yuxia Jian, Xue Wang, Lei Pan, Wen Guo

**Affiliations:** Shandong Key Laboratory of Disease Control in Mariculture, Key Laboratory of Benthic Fisheries Aquaculture and Enhancement, Marine Science Research Institute of Shandong Province (National Oceanographic Center, Qingdao), Qingdao, 266104 China

**Keywords:** Genome, Genetics

## Abstract

Fat greenling (*Hexagrammos otakii* Jordan & Starks, 1895) is a valuable marine fish species, crucial for aquaculture in Northern China due to its high-quality meat and significant economic value. However, the aquaculture industry faces challenges such as trait degradation, early sexual maturity, and disease susceptibility, necessitating advanced genomic interventions for sustainable cultivation. This study presents the first chromosomal-level genome assembly of *H. otakii*, achieved using PacBio long-read sequencing and Hi-C technology. The assembly yielded a genome size of 682.43 Mb with a contig N50 size of 2.39 Mb and a scaffold N50 size of 27.83 Mb. The completeness of genome assessed by BUSCO is 96.99%. A total of 22,334 protein-coding genes were predicted, with 21,619 (96.80%) functionally annotated across various protein databases. This genomic resource is a step forward in supporting the breeding, germplasm conservation, and enhancement of *H. otakii*, facilitating genetic studies and the development of strategies for disease resistance and growth optimization in aquaculture.

## Background & Summary

**Fig. 1:**
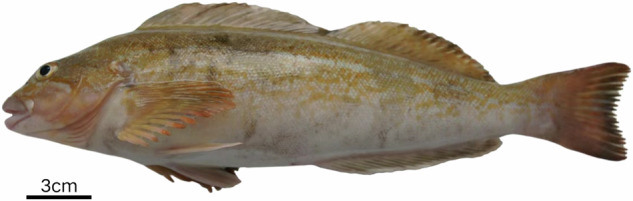
A picture of fat greenling (*Hexagrammos otakii*) used for the genome sequencing.

Fat greenling (*H. otakii*) is a cold-water near-bottom reef fish (Fig. 1), mainly distributed in northern China such as Shandong, Hebei, Tianjin, Liaoning provinces. It also inhabits the seas of North Korea, Japan and the Far East of Russia^1–4^. Renowned for its high-quality meat and nutritional value, fat greenling is a cornerstone species in marine economic fisheries and is highly valued by both consumers and farmers. It is an ideal candidate for cage culture, proliferation, release, and resource restoration, with significant potential for broader application in aquaculture across Northern China. *H. otakii* prefers coastal and rocky reef habitats, typically found at depths up to 50 meters. This species is resilient to low temperatures, thriving in waters ranging from 2 to 26 °C, with an optimal growth temperature between 16 and 21 °C. The salinity range suitable for its survival is 16 to 32‰, allowing it to overwinter safely in northern sea areas^5^. As an omnivorous species, its diet is diverse, including over 40 different items such as shrimp, fish, clams, worms, and amphipods.

Reproduction in fat greenling is characterized by a single annual spawning event from mid-to-late October to late November, triggered when water temperatures drop to around 18 °C. Female fat greenling produce a relatively small number of eggs, typically between 2,000 and 9,000. Eggs from sexually mature female fishes (about 150 mm in length) range from 2,000 to 3,000, while the older females (aged 4-5 years and over 250 mm in length) can produce between 6,000 and 9,000 eggs. However, the wild population of *H. otakii* has been declining due to overfishing, nitrogen and phosphorus pollution, and increased industrial energy consumption, which has necessitated urgent measures to repair and conserve fishery resources. The development of fat greenling aquaculture has drawn significant attention due to these challenges. The main issues hindering the development and scaling of its market include the limited availability of resources and challenges in large-scale artificial seedling production, such as early sexual maturity, reduced ovarian egg production, and extended hatching periods. These issues create a bottleneck in the artificial breeding process.

The artificial breeding of fat greenling faces specific challenges due to the high viscosity of the eggs, which leads to the formation of agglomerates during artificial insemination and results in extremely low fertilization rates. Moreover, the prolonged incubation time of fertilized eggs renders them highly susceptible to pathogens, further causing hatching difficulties. Before the breakthrough in artificial breeding techniques, wild fry was used for cage culture, leading to instability in fry yields and a downward trend that could not meet the demands of high-volume production. Furthermore, harvesting wild seed contradicts the principles of fishery resource restoration and ecological protection.

The breakthrough in artificial breeding has alleviated the supply issues of fries, leading to an increase in cage breeding and marine stock enhancement and release, with a gradual recovery of natural resources. With the rapid development of fat greenling industry, the lag of breeding has been highlighted. Most of the fries for cage culture and marine stock enhancement and release come from the offspring of artificially bred wild broodstock. Issues such as lodging syndrome, skin ulcer disease, rotten tail disease, and bacterial rotten gill disease occur during the breeding process, with the underlying causes still unclear but potentially linked to genetic factors like individual physical disabilities, developmental deficiencies, and compromised immunity^6^.

Given the mixed germplasm, unknown parental history, and trait degradation, the primary challenges for fish fry include slow growth rate, weak disease resistance, and high mortality. High-quality and sufficient seed has become a key factor in the industrial chain of fat greenling. The current breeding situation highlights that it is urgent to conduct genome research to obtain genome sequence and study its immune regulation mechanism. It is worthwhile to conduct researches on wild population conservation and genetic improvement of fat greenling.

At present, the research on the fat greenling involves many aspects, including embryonic development^7^, genetic diversity^8–10^, nutritional requirements^11–13^, Breeding environmental conditions^14–19^, immunology^20–22^, behavioristics^23,24^ and so on. However, genomic resources for this species are limited, with only transcriptome and microsatellite marker analyses available, and no reference genome exists. This lack of genomic data hampers conservation and genetic studies of the species.

Advancements in sequencing technologies have paved the way for genome information acquisition^25^ and molecular breeding^26^, proving to be valuable methods. In this study, the chromosome-level genome of fat greenling was firstly constructed using PacBio sequencing and Hi-C technology. A 350 bp library was constructed and generated a total of 73.5 Gb of clean data on an Illumina HiSeq platform (Tables 1 and 2). The PacBio platform produced 113.58 Gb of high-quality clean reads, representing a 174.74-fold coverage of the fat greenling genome (Tables 1 and 4). This genomic data will benefit comprehensive conservation studies of fat greenling to implement better protection of wild populations, and it will facilitate researches on population genetics and the identification of functional genes related to important economic traits and the sex determination for fat greenling. Additionally, this data will provide crucial theoretical guidance for artificial and genetic engineering breeding, aiding in the screening of genetic variations related to rapid growth and disease resistance.

**Table 1:** Statistics for the sequencing data of fat greenling genome.

The statistics of Illumina short read data					
Read number	Base count (Gb)	Read length (bp)	Q20 (%)	Q30 (%)	GC_content (%)
493,072,248	73.5	150	96.64	91.37	43.25

**Table 2:** The statistics of Illumina short read data.

The statics for the genome survey (K-mer = 17)					
K-mer number	K-mer depth	Genome size (Mb)	Revised genome size (Mb)	Heterozygous ratio (%)	Repeat (%)
57,249,936,244	80	689	678	0.70	37.52

## Methods

### Sample collection and DNA extraction

To obtain high-quality genomic DNA for sequencing, the fresh muscle tissue was collected from a fat greenling in Lidao, Rongcheng city, Weihai city, Shandong province. The muscle tissue below the dorsal fin was taken and stored in liquid nitrogen until DNA extraction. The improved CTAB (Cetyltrimethylammonium bromide) method was used to extracted high-quality genomic DNA. The quality and concentration of the extracted genomic DNA were checked using NanoDrop 2000 spectrophotometer (NanoDrop Technologies, Wilmington, DE, USA), 0.8% agarose gel electrophoresis and a Qubit 3.0 fluorimeter (Life Technologies, Carlsbad, CA, USA).

### Library construction, sequencing and data preparation

The second-generation DNA fragment library, PacBio CLR library and Hi-C data were obtained for generating a chromosome-level genome assembly of fat greenling. 1 μg DNA was used to construct the library according to the MGI DNA Library Kit (Vazyme, Nanjing) protocol. Then, the concentration and fragment size distribution of the samples in the constructed library were determined by Qubit 3.0 fluorometer (Life Technologies, Carlsbad, CA, USA) and Bioanalyzer 2100 system (Agilent Technologies, CA, USA) using the appropriate computer pooling program. After library validation, sequencing was performed on the MGISEQ-2000 platform (the sequencing service is provided by Wuhan Onemore Technology Co., Ltd.). A 350 bp library was constructed and generated a total of 73.5 Gb of clean data for the fat greenling on an Illumina NovaSeq 6000 platform, with Q20 and Q30 being 96.64% and 91.37% respectively (Tables 1 and 2). The SMRTbell Express Template Prep Kit 2.0 reagent (Pacific Biosciences) were used to constructed the SMRT Bell CLR Library. Approximately 5 μg of genomic DNA was used for the library construction. The library size and quality were evaluated using FEMTO Pulse and Qubit dsDNA HS assay kits. Sequencing primers and Sequel II DNA polymerases were annealed separately and combined with the final SMRTbell library. After library construction, sequencing was performed on the PacBio Sequel II platform (the sequencing service is provided by Wuhan Onemore Technology Co., Ltd.). In total, 113.58 Gb of clean data were obtained, which was 174.74-fold coverage of the genome assembly. Hi-C libraries were constructed using MboI restriction enzyme and sequenced on the Illumina NovaSeq 6000 platform in 150 bp paired-end mode. As a result, 101.7 Gb of Hi-C clean data were obtained, which covered 156.46-fold of the gene assembly (Table 1).

Additionally, muscle, heart, spleen, liver and stomach tissues were pooled to obtain the transcriptome of fat greenling. Transcriptome sequencing was performed on the Illumina NovaSeq 6000 platform, yielding a total of 7.8 G of clean data (Table 1).

### Genome survey

**Fig. 2:**
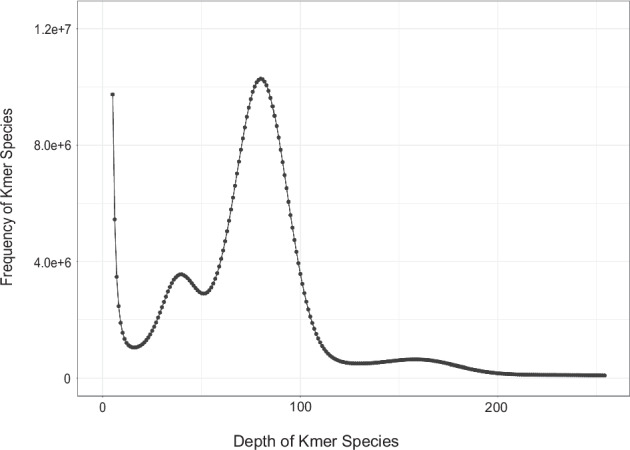
Distribution of k-mers of length 17 from the Illumina data.

After obtaining the second-generation sequencing data PE150, HTQC v1.92.310 software^27^ was used to filter the raw data to obtain high-quality data. A survey of the fat greenling genome was performed using the k-mer (k = 17 in this case) method to estimate genome size, heterozygosity and repeat sequence information The software GCE(1.0.0)^28^ was utilized, and the total number of k-mers was 57,249,936,244, with a k-mer peak at a depth of 80 (Table 3, Fig. 2). the fat greenling genome size was estimated to be 689 Mb, revised to 678 Mb. The heterozygous ratio was 0.70%, and the repetitive rate was 37.52% (Table 3).

**Table 3:** The statics for the genome survey (K-mer = 17).

Total number	Total number (>2 kb)	Total bases (Gb)	Max length (bp)	Mean length (bp)	N50 (bp)	N90 (bp)	GC Content (%)
8,702,531	7,808,953	113.58	140,318	13,051	20,367	7,160	43.20

### De novo genome assembly

**Fig. 3:**
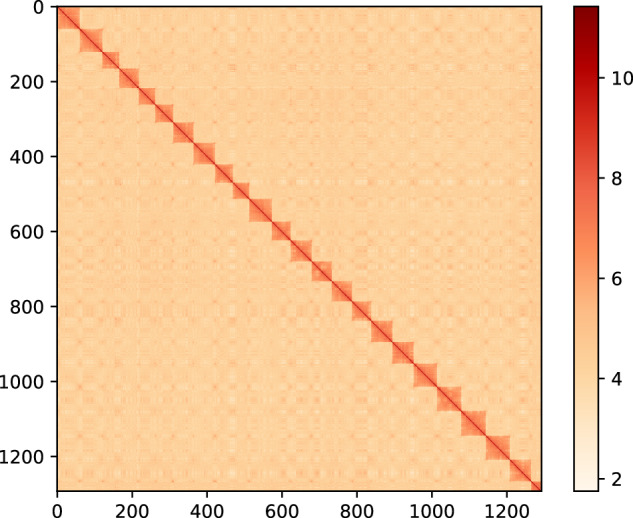
Chromosomal Hi-C heatmap of the *H. otakii* genome assembly.

Genome assembly was performed by PacBio Sequel II CLR sequencing mode to obtain subreads data. 100 × random data were used for preliminary assembly by Mecat2 software^29^ with default parameters. After the initial assembly, the gcpp tool of SMRT Link v8.0 (https://github.com/PacificBiosciences/gcpp) was used to perform the third-generation error correction, and then used the second-generation data in the survey evaluation to perform the genome second-generation polish. After the polish was completed, we completed genome assembly at the contig level.The PacBio platform generated a total of 113.58 Gb high-quality clean reads from the long-read library, representing a 174.74-fold coverage of the fat greenling genome (Tables 1 and 4). These data were assembled using Canu(v2.2)^30^, followed by polishing with Plion(v1.22)^31^. For highly heterozygous genomes, initial assembly may assemble all the heterozygous fragments, resulting in larger genomes than expected. Purge_haplotigs(v1.0.4)^32^ was used to remove redundancy in the genome after initial assembly error correction. Redundant contigs were identified and removed according to the depth distribution and sequence similarity of reads. The genome fragment of 682.34 Mb was obtained, the length of which was close to the genome size estimated by k-mer analysis. The clean Hi-C data were aligned to the genome assembly using BWA (v0.7.12)^33^. During the Hi-C assisted assembly and error correction process, the original 1,026 contigs were broken and sorted based on the interaction map, and finally 24 chromosomes and 469 scaffolds were constructed. The sequence and direction of the *H. otakii* genome were determined. The final total length of the genome was 682.43 Mb, with a contig N50 of 2.39 Mb, scaffold N50 of 27.83 Mb, and a chromosome anchoring rate of 94.69% (Fig. 3; Tables 5 and 6). The genome size of *H. otakii* was smaller than that of *Hexagrammos agrammus*^34^.

**Table 4:** The Pacbio subreads used for genome assembly.

Mode	Total length (bp)	Total number	Total number (>=2 kb)	Max length (bp)	N50 (bp)	N90 (bp)	GC content (%)
Assembly	1,057,810,994	6,149	6,088	18,729,851	683,948	58,704	43.60
Assembly + arrow	1,058,530,173	6,149	6,088	18,743,424	684,330	58,716	43.60
Assembly + arrow + polion	1,058,207,179	6,149	6,088	18,736,102	684,205	58,715	43.60
Polish + Purge_haplotigs	745,651,162	2,071	2,056	18,736,102	1,703,659	135,237	43.20
Polish + Purge_haplotigs + DelPollution	682,337,610	1,026	1,017	18,736,102	2,410,640	294,676	43.00

**Table 5:** Statistics of the fat greenling assembly before Hi-C correction.

	Sequence length (bp)	Sequence number	Contig N50 (bp)	Scaffold N50 (bp)	Contig number (%)	Contig length (%)	Contig > 100 kb number (%)	Contig > 100 kb length (%)
raw	682337610	1026	2410640	2410640	—	—	—	—
hi-c	682426410	493	2391995	27834403	52.21	94.69	86.09	97.46
hi-c chr	646186390	24	2846330	28148260	—	—	—	—
hi-c nochr	36240020	469	93906	134000	—	—	—	—

**Table 6:** Hi-C_statistics of *Hexagrammos otakii*.

Mapping Rate (%)	Average	Coverage (%)	Coverage at least 4X(%)	Coverage at least 10X(%)	Coverage at least 20X(%)
	sequencing depth				
98.37	35.94	99.76	99.02	94.49	80.12

### Assessment of the genome assemblies

To assess the completeness and accuracy of the genome assembly, Minimap2 (the default parameter of V2.5)^35^ was employed to align the CLR data from the third generation sequencing back to the assembled genome. The alignment rate, coverage degree and depth distribution of reads were counted to evaluate the integrity of assembly and uniformity of sequencing coverage. BWA software^28^ was used to map the second-generation sequencing data back to the assembled genome. The rate of successfully aligned reads was recorded to assess the quality of the assembly. Then GATK software^36^ was used to conduct SNP calling and filtering, and the results of SNPs (statistical heterozygous and homozygous) and InDels were analyzed. The Benchmarking Universal Single-Copy Orthologs (BUSCO v3.0.2)^37^ analysis was conducted to evaluate the assembly completeness. Using the OrthoDB database, BUSCO(actinopterygii_odb9) predicted the single-copy homologous genes and calculated their integrity, fragmentation, and possible loss rate. This assessment provided a quantitative measure of the integrity of the gene regions in the entire assembly.

The Illumina reads and PacBio-long reads were aligned using the software BWA and BLAST^38^ to evaluate the quality of initial assembly. The results showed that 99.27% of the Illumina reads and 98.37% of the PacBio long reads were successfully mapped to the assembled genome (Tables 6 and 7). This high mapping rate indicates a high-quality initial assembly.

**Table 7:** The alignment of the Pacbio subreads to the fat greenling genome assembly.

Type	Repeat Size	% of genome
Trf	93,669,836	13.73
Repeatmasker	100,080,051	14.67
Proteinmask	16,645,016	2.44
De novo	244,182,803	35.79
Total	301,301,969	44.16

### Repeat sequence annotation

Both homology annotation and *de novo* annotation were used to identify repetitive sequences in the genome. Firstly, RepeatMasker (open-4.09)^39^ and RepeatProteinMask (open-4.09) were used to search for transposable element (TE) sequences from the Repbase (release 21.01)^40^ database based on homology. Secondly, RepeatModeler (open-1.0.11)^41^ and LTR-FINDER (v1 .0.5) Software^42^ were used to construct a repetitive sequence database of species from scratch. RepeatMasker (open-4.09) was utilized to identify repetitive sequences from the constructed database. In addition, Tandem Repeats Finder (TRF)^43^ was used to identify tandem repetitive sequences.

The results from both homology and *de novo* annotation methods were integrated, removing overlapping non-redundant parts to finalize the repeat sequence annotation. A total of 301.3 Mb of repeat sequences were detected, accounting for 44.16% of the assembly genome (Table 8). This repeat content was larger than the value (37.52%) obtained from k-mer analysis. The predominant repeats type was DNA (203.08 Mb; 29.76% of assembly), LINEs (65.5 Mb; 9.60%), and LTRs (42.2 Mb; 6.18%) (Table 9).

**Table 8:** Statistics of repetitive sequences in the fat greenling.

Type	Length (Bp)	% in genome	Length (Bp)	% in genome	Length (Bp)	% in genome	Length (Bp)	% in genome
DNA	66297392	9.72	3027360	0.44	153801656	22.54	203084087	29.76
LINE	20190728	2.96	9864976	1.45	53012281	7.77	65501248	9.60
SINE	4158128	0.61	0	0	3455517	0.51	6889820	1.01
LTR	16190027	2.37	3759085	0.55	28533170	4.18	42195939	6.18
Satellite	5525756	0.81	0	0	6704413	0.98	11887072	1.74
Simple_repeat	0	0	0	0	8732125	1.28	8732125	1.28
Other	3992	0	63	0	0	0	4055	0
Unknown	732739	0.11	10629	0	20555047	3.01	21285979	3.12
Total	100080051	14.67	16645016	2.44	228746265	33.52	278406157	40.80

**Table 9:** Statistics on transposable elements in fat greenling genome.

Gene set	Number	Average gene length (bp)	Average CDS length (bp)	Average exon per gene	Average exon length (bp)	Average intron length (bp)
denovo/Genscan	24503	17459.73	1502.18	8.58	175.12	2105.78
denovo/AUGUSTUS	30979	9989.96	1210.22	6.65	181.92	1553.29
homo/Cottoperca_gobio	54518	15079.1	1080.6	5.81	185.93	2909.18
homo/Acanthochromis_polyacanthus	59261	13618.43	1038.31	5.65	183.88	2707.27
homo/Amphiprion_ocellaris	56730	14099.34	1073.63	5.84	183.7	2688.84
homo/Notothenia_coriiceps	70056	12181.74	856.79	4.52	189.68	3220.07
homo/Perca_flavescens	59831	14392.92	1055.29	5.65	186.66	2866.19
trans.orf/RNAseq	11608	15538.57	1791.56	11.66	306.43	1121.96
BUSCO	4651	11838.65	1954.86	12.48	156.65	861
MAKER	22413	14228.57	1518.54	9	252.4	1493.88
HiCESAP	22334	14477.64	1676.93	10.07	255.04	1312.62

### Coding gene structure and function annotation

Homologous annotation, *de novo* annotation and transcriptome assisted annotation were used to predict the structure and function of coding genes.

Related species at the Order level including *Cottus gobio*, *Notothenia coriiceps*, *Perca flavescens*, *Acanthochromis polyacanthus*, and *Amphiprion ocellaris* were selected as homolog for protein-coding gene annotation. TblastN software^44^ was used to compare protein sequences from these species to the *H. otakii* constructed reference genome. Following sequence comparison, the corresponding query proteins and their sequences were filtered and processed using Exonerate software^45^, Augustus (v3.3)^46^, Genscan, and GlimmerHMM (V3.0.4) to initiate gene structure annotations. For the second-generation transcriptome data (RNA-Seq), Tophat software^47^ was used to align the RNA-Seq reads to the constructed reference genome. The aligned sequences were then assembled into gene structures using Cufflinks software^48^. The open reading frames (ORFs) in the transcript were predicted using TransDecoder software^49^ to define the putative coding sequence (CDS). MAKER (V3.00) software^50^ integrated the gene sets predicted by homologous, *de novo*, and transcriptome-assisted methods into a comprehensive, non-redundant gene set. List of protein function databases (SwissProt, TrEMBL, KEGG, InterPro, GO, AnimalTFDB, etc.) were used to annotate the annotated gene set with information on protein function, metabolic pathways, structural domains, and other gene functions and metabolic pathways.

**Fig. 4:**
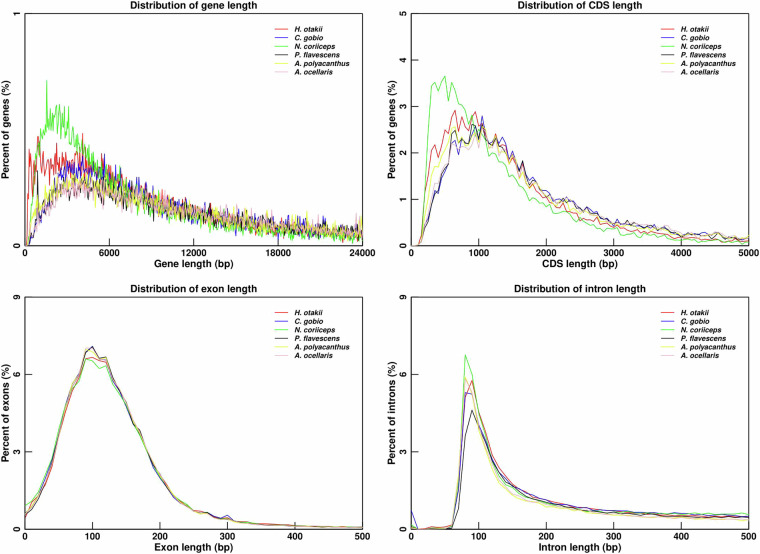
Annotation quality comparison of protein-coding genes. The gene length, CDS length, exon length, and intron length were compared among 6 species: *Hexagrammos otakii*, *Cottoperca gobio*, *Acanthochromis polyacanthus*, *Amphiprion ocellaris*, *Notothenia coriiceps*, *Perca flavescens*.

**Fig. 5:**
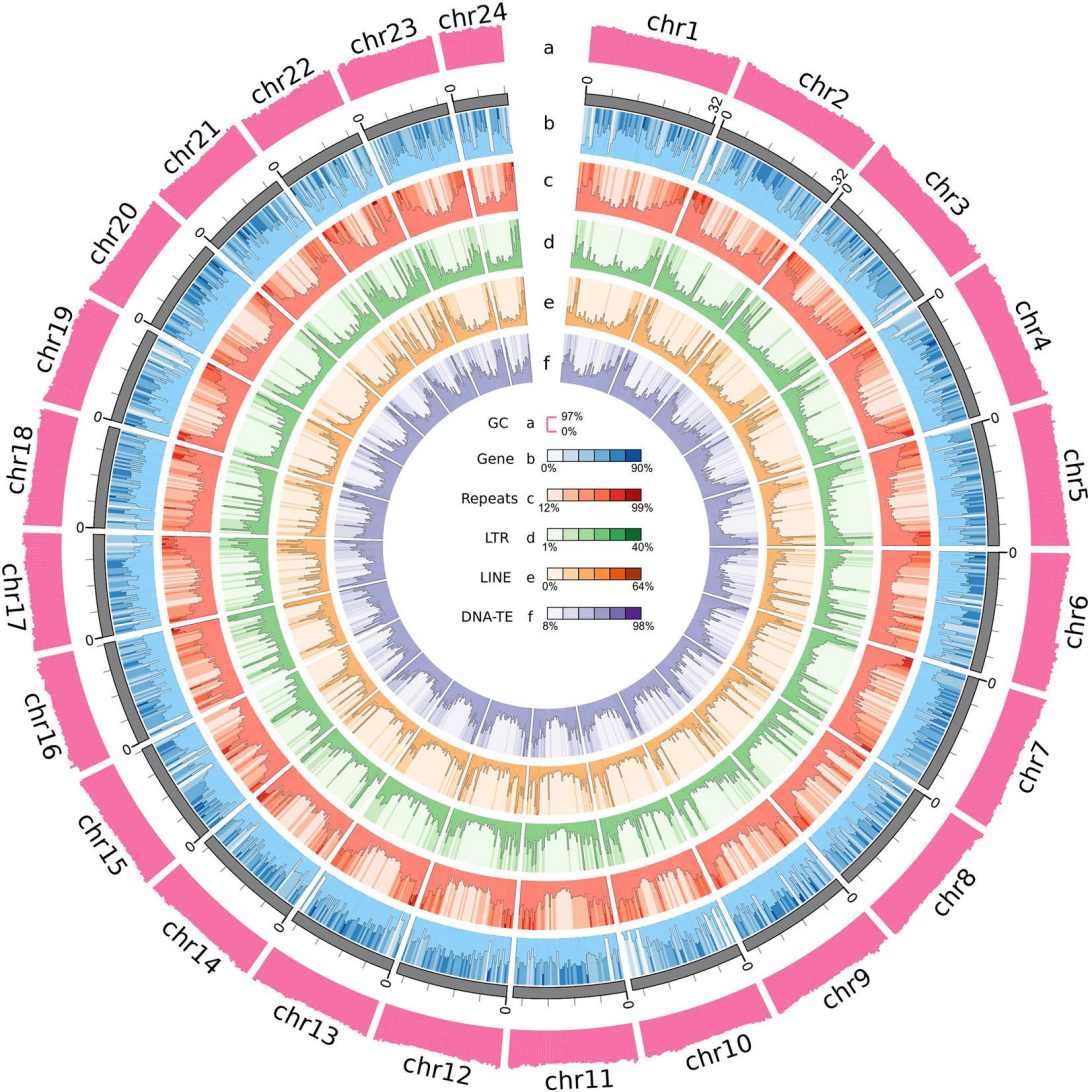
Genome characteristics of fat greenling.

Overall, these combined approaches predicted 22,334 protein-coding genes, with average gene, exon, and intron lengths of 14,477.64 bp, 255.04 bp, and 1,312.62 bp, respectively (Table 10). The statistics of the predicted gene models were compared with those from five other teleost species, including *C. gobio*, *A. polyacanthus*, *A. ocellaris*, *N. coriiceps*, and *P. flavescens*, showing similar distribution patterns in mRNA length, CDS length, exon length, and intron length (Fig. 4). The summary of the genome characteristics of fat greenling is shown in Fig. 5. A total of 21,619 genes, accounting for 96.80% of the predicted genes, were successfully annotated by alignment to the nucleotide, protein, and annotation databases including InterPro, NR, Swissprot, TrEMBL, KOG, GO, and KEGG (Table 11).

**Table 10:** Statistics of gene predictions in the fat greenling genome.

Annotation database	Annotated number of predicted genes	Percent (%)
InterPro	19830	88.79
GO	15081	67.52
KEGG_ALL	21383	95.74
KEGG_KO	14130	63.27
Swissprot	19343	86.61
TrEMBL	21473	96.14
TF	3383	15.15
Pfam	19048	85.29
NR	21552	96.50
KOG	17815	79.77
All annotated	21619	96.80
Predicted genes	22334

**Table 11:** Summary of functional annotations for predicted genes.

Type		Copy	Average length (bp)	Total length (bp)	% of genome
miRNA		707	86	60583	0.008878
tRNA		4942	75	371929	0.054501
rRNA	rRNA	3409	122	414482	0.060737
	18S	9	1170	10529	0.001543
	28S	0	0	0	0
	5.8S	4	144	576	0.000084
	5S	3396	119	403377	0.059109
snRNA	snRNA	718	138	98799	0.014478
	CD-box	225	135	30480	0.004466
	HACA-box	76	149	11328	0.00166
	splicing	410	135	55352	0.008111
	scaRNA	7	234	1639	0.00024

### Non-coding RNA annotation

In the process of non-coding RNA annotation, tRNAscan-SE (V1.3.1) software^51^ was used to search for tRNA sequences in the genome according to the structural characteristics of tRNA. Since rRNA is highly conserved, rRNA sequences of closely related species were selected as reference sequences to search for rRNA in the genome by BLASTN (V2.6.0) alignment. In addition, Rfam (V14.1) family covariance model was used to predict miRNA and snRNA sequence information on the genome by INFERNAL software^52^ of Rfam. A total of 4,942 tRNAs, 3,409 rRNAs, and 707 microRNAs were identified by non-coding RNA prediction (Table 12).

**Table 12:** Statistics of the noncoding RNA in the fat greenling genome.

	Proteins	Assembly Percentage (%)	Proteins	Annotation Percentage (%) Percentage (%)
Complete BUSCOs	4442	96.90	4419	96.40
Complete Single-Copy BUSCOs	4308	94.00	4245	92.60
Complete Duplicated BUSCOs	134	2.90	174	3.80
Fragmented BUSCOs	50	1.10	71	1.50
Missing BUSCOs	92	2.00	94	2.10
Total BUSCO groups searched	4584	100	4584	100

## Data Records

The raw data, including Illumina and Hi-C sequencing data have been deposited in a public database. Hi-C data of *Hexagrammos otakii*: SRR27636748^53^, Pacbio subreads data of *Hexagrammos otakii*: SRR27636749^54^, Whole Genome Sequencing (WGS) data of *Hexagrammos otakii*: SRR27636750^55^, RNA-seq data of *Hexagrammos otakii*: SRR31132309^56^, Assembly data of *Hexagrammos otakii*: GCA_043790635.1^57^, Annotation data of *Hexagrammos otakii*: 10.6084/m9.figshare.27299841^58^.

## Technical Validation

### Evaluating quality of DNA and RNA

Prior to the genome sequencing, we used an improved CTAB method to extracted high-quality genomic DNA. The quality and concentration of the extracted genomic DNA was checked using NanoDrop 2000 spectrophotometer (NanoDrop Technologies, Wilmington, DE, USA), 0.8% agarose gel electrophoresis and a Qubit 3.0 fluorimeter (Life Technologies, Carlsbad, CA, USA).

### Evaluating quality of genome assembly

The Illumina reads and PacBio long reads were aligned using BWA and BLAST to evaluate the quality of initial assembly. The results showed that 99.27% of the Illumina reads and 98.37% of the PacBio long reads were successfully mapped to the assembled genome (Tables 6 and 7). BUSCO analysis was conducted to evaluate the assembly quality based on the OrthoDB database. A total of 96.90% of the 4,584 single-copy orthologs in the assembled genome were determined as complete, including 4,308 single-copy (94.00%) and 134 duplicated (2.90%) orthologs, 1.10% and 2.00% of the total single-copy orthologs were fragmented and missing, respectively (Table 13).

**Table 13:** Results of BUSCO analysis of the fat greenling genome.

Type	Sequencing technology	Sequencing platform	Library Size(bp)	Clean Data(Gb)	Coverage^a^
Genome	Illumina	Illumina NovaSeq 6000	350	73.5	113.08
Genome	PacBio	PacBio Sequel II	20000	113.58	174.74
Genome	Hi-C	Illumina NovaSeq 6000	350	101.7	156.46
RNA	Illumina	Illumina NovaSeq 6000	350	7.8 G	

### Evaluating quality of the genome annotation

BUSCO analysis was conducted to evaluate the genome annotation quality based on the OrthoDB database. A total of 96.40% of the 4,854 single-copy ortholog genes in the assembled genome were determined as complete, including 4,245 single-copy genes (92.60%) and 174 duplicated genes (3.80%), 1.50% and 2.10% of the total genes were fragmented and missing, respectively (Table 13).

## Data Availability

No specific code was used in this study. The commands used in the processing were all executed according to the manuals and protocols of the corresponding bioinformatics software.
